# Written communication for itu discharges: a quality improvement project

**DOI:** 10.1186/2197-425X-3-S1-A143

**Published:** 2015-10-01

**Authors:** D Bendel, H Gardner, M Outram

**Affiliations:** Northampton General Hospital, Department of Anaesthesia and Critical Care, Northampton, United Kingdom

## Introduction

ITU patients are complex, and detailed written handover in the form of a discharge letter plays a central role in patient safety[1]. We sought to characterise and improve upon the quality of our written handover on discharge.

## Objectives

To improve the quality of written discharge communication.

## Methods

In 2014, we audited our discharge summaries over a two month period. Our adopted standards were the National Institute of Health and Clinical Excellence (NICE) clinical guidelines 50 and 83[2]^,^[3]. They detail various domains that should be present on a summary. These domains formed the basis of our audit proforma.

Discharge summaries were then scored according to whether the domains were satisfied or not. The total score for each domain was then divided by the total number of discharge summaries, in order to indicate the percentage of summaries that satisfied each domain.

**Figure 1:**
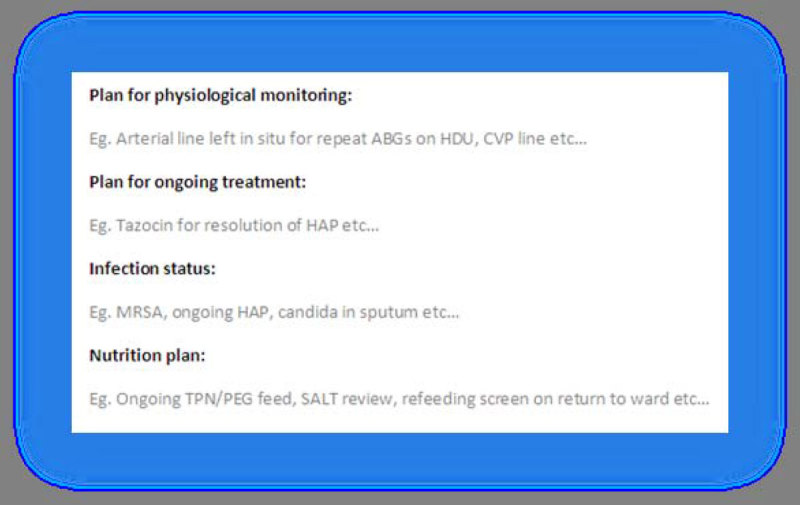
**Detail of the new discharge summary.**

Following this audit, we introduced an electronic discharge summary template. The template is composed of mandatory headings that cannot be deleted. Each heading addresses a different domain from NICE guidance, and is followed by a free-text box. They contain suggestions in grey that disappear upon entering information (Figure 1).

We closed the audit loop in 2015.

## Results

Our discharge summaries fell short of our adopted standards across several domains. Frequent omissions included legal aspects such as patient demographics (10%), Dr's designation (8%) and bleep number (68%). Other regularly omitted domains included a plain-English summary for relatives (18%), relevant investigations (35%) and a plan for patient monitoring on return to the ward (45%).

This was due, at least in part, to the document's 'free-text' nature.

**Figure 2:**
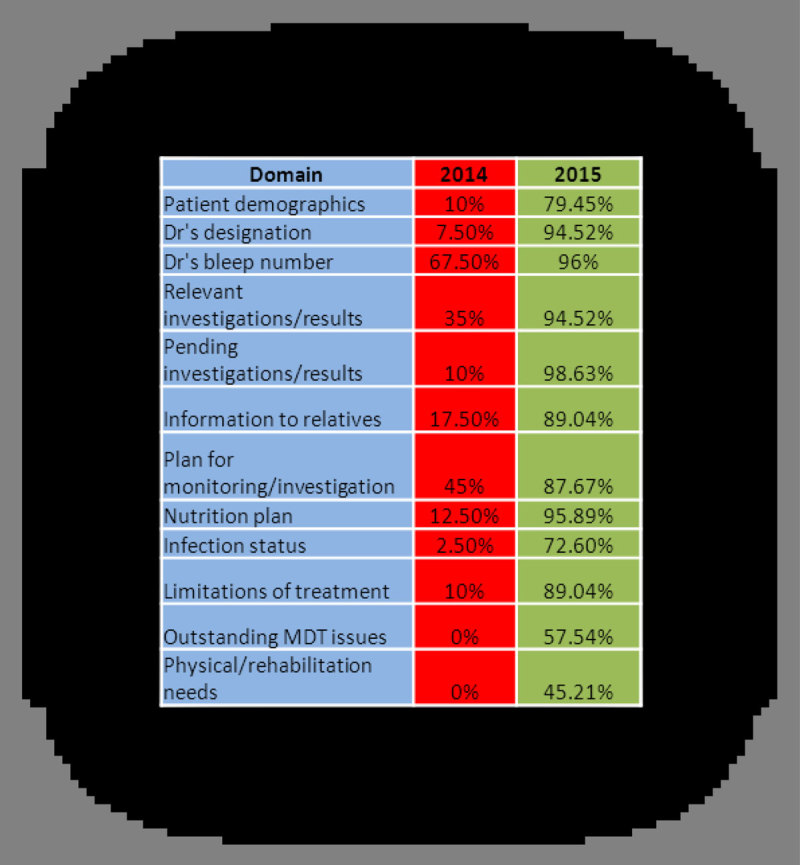
**Areas of significant improvement**

On re-audit, the legal aspects of our summaries are almost 100% compliant. There is improvement to areas such as reason for admission (99%) and diagnosis (85%). New information that now features on our summaries includes pending investigations, nutrition plan, infection status, limitations of treatment and information for relatives (figure 2).

Our new discharge template has dramatically improved the quality of our written handovers.

## Conclusions

The safe handover of ITU patients relies on detailed written communication, in line with NICE guidance. Simple technological interventions such as the discharge template described here are an effective way of achieving this.

## References

[1] van Sluisveld N, Hesselink G, van der Hoeven JG, Westert G, Wollersheim H, Zegers M (2015). Improving clinical handover between intensive care unit and general ward professionals at intensive care unit discharge. Intensive Care Medicine.

[2] National Institute for Health and Clinical Excellence (NICE) (2007). CG50 Acutely ill patients in hospital: NICE guideline.

[3] National Institute for Health and Clinical Excellence (NICE) (2009). CG83 Critical Illness Rehabilitation: NICE Guideline.

